# Improving treatment of patients with psychosis in low-and-middle-income countries in Southeast Europe: Results from a hybrid effectiveness-implementation, pragmatic, cluster-randomized clinical trial (IMPULSE)

**DOI:** 10.1192/j.eurpsy.2022.2302

**Published:** 2022-08-10

**Authors:** N. Jovanović, M. Russo, T. Pemovska, J. J. Francis, A. Arenliu, S. Bajraktarov, A. Džubur Kulenović, L. Injac Stevović, A. Novotni, S. Andrić Petrović, T. Radojičić, E. Ribić, J. Konjufca, N. P. Marić

**Affiliations:** 1 Wolfson Institute of Population Health, Queen Mary University of London, United Kingdom; 2 Melbourne School of Health Sciences, The University of Melbourne, Parkville, Victoria, Australia; 3 Department of Psychology, University of Prishtina, Pristina, Kosovo* by United Nations resolution; 4 Medical Faculty, University “Ss. Cyril and Methodius”, University Clinic of Psychiatry, Skopje, North Macedonia; 5 Department of Psychiatry, Clinical Center University of Sarajevo, Sarajevo, Bosnia and Herzegovina; 6 Psychiatric clinic, Clinical Centre of Montenegro, Podgorica; Medical faculty, University of Montenegro, Montenegro; 7 Faculty of Medicine, University of Belgrade, Belgrade, Serbia

**Keywords:** Digital, implementation, mental health, psychosis, randomized controlled trial

## Abstract

**Background:**

In Southeast Europe (SEE) standard treatment of patients with psychosis is largely based on pharmacotherapy with psychosocial interventions rarely available. DIALOG+ is a digital psychosocial intervention designed to make routine care therapeutically effective. This trial simultaneously examined effectiveness of DIALOG+ versus standard care on clinical and social outcomes (Aim 1) and explored intervention fidelity (Aim 2).

**Methods:**

A hybrid type II effectiveness–implementation, cluster-randomized trial was conducted in five SEE countries: Bosnia and Herzegovina, Kosovo*, Montenegro, North Macedonia, and Serbia. The intervention was offered to patients six times across 12 months instead of routine care. The outcomes were subjective quality of life (primary), clinical symptoms, satisfaction with services, and economic costs. Intervention fidelity was operationalized as adherence to the protocol in terms of frequency, duration, content, and coverage. Data were analyzed using multilevel regression.

**Results:**

A total of 81 clinicians and 468 patients with psychosis were randomized to DIALOG+ or standard care. The intervention was delivered with high fidelity. The average number of delivered sessions was 5.5 (SD = 2.3) across 12 months. Patients in the intervention arm had better quality of life (MANSA) at 6 months (*p* = 0.03). No difference was found for other outcomes at 6 months. Due to disruptions caused by the COVID-19 pandemic, 12-month data were not interpretable.

**Conclusions:**

DIALOG+ improved subjective quality of life of individuals with psychosis at 6 months (after four sessions), albeit with small effect size. The intervention has the potential to contribute to holistic care of patients with psychosis.

## Introduction

Over 200 million people worldwide experience psychotic disorders such as schizophrenia, schizoaffective, and bipolar affective disorder [1]. These disorders have a number of serious symptoms, such as delusional thinking, perceptual abnormalities, low or elated mood, and poor social functioning [1]. Healthcare systems in high-income countries provide a combination of care, medication, and psychosocial interventions which help a number of people to lead a productive life [2, 3].

A recent evaluation of mental health care provided to people with psychosis in Central and Eastern Europe suggests that mental health care across the region remains based around psychiatric hospitals and is limited to prescription of medications [4]. However, medication alone may not lead to complete resolution of symptoms in at least one-third of patients [5]. The low- and middle-income countries (LMICs) in Southeast Europe (SEE) may not be able to provide a holistic approach (i.e., pharmacological and psychosocial interventions) to patients with psychosis due to limited human and financial resources [6, 7]. Mental health services need effective, low-cost, and easily deliverable psychosocial interventions to expand access to care for the benefit of these patients and their families.

DIALOG+ is a digital psychosocial intervention based on patient-centered communication theories, quality of life research, advances in information technology, and principles of solution-focused therapy [8–10]. It was specifically designed to make routine clinical appointments between clinicians and patients with psychosis therapeutically effective [8]. The intervention costs include clinician training (3 h), supervision (up to 3 h spread across a few months as per each clinician’s need), and tablet computer. Using the intervention does not prevent the use of other treatments at the same time. DIALOG+ has been shown to be effective and cost-effective for patients with psychosis in the UK [8]. In a cluster-randomized controlled trial (cRCT), it was used monthly and compared to an active control. After 1 year, patients in the intervention arm had better subjective quality of life, fewer unmet needs, lower general symptom levels, and better social outcomes, as well as lower treatment costs [8]. The DIALOG+ intervention was associated with a cost saving of GBP 1,288 after controlling for baseline costs [8]. This finding can be considered as a facilitator for the implementation and sustainability of the intervention from the point of healthcare services.

This study had two aims: *Aim 1 (effectiveness)* was to explore the clinical effectiveness and cost-effectiveness of the DIALOG+ intervention relative to standard care on patient-level clinical and social outcomes in five LMICs in Southeast Europe. *Aim 2 (implementation)* was to explore the intervention fidelity, defined as the degree to which an intervention is delivered as intended. A conceptual framework developed by Carroll et al. was used to explore intervention fidelity through adherence to the study protocol in terms of frequency, duration, content, and coverage [11]. The DIALOG+ intervention was implemented in services based on the implementation strategy developed before the trial (see section “Methods”).

## Methods

### Study design

This is a multicountry, pragmatic, hybrid type II effectiveness–implementation, cluster-randomized, clinical trial (ISRCTN 11913964). Clusters were clinicians working in outpatient mental health services in five countries: Bosnia and Herzegovina, Kosovo*, Montenegro, North Macedonia, and Serbia. Clinicians were randomly assigned to either DIALOG+ (intervention) or standard care (control). The intervention arm received DIALOG+ during routine clinical appointments instead of usual care. The control arm received usual care during also routine clinical appointments. A cluster randomization design was used to avoid potential contamination of the practice by clinicians when treating patients in both arms. The trial explored effectiveness, cost-effectiveness and fidelity of the DIALOG+ intervention relative to standard care. Detailed description of methods can be found in the study protocol [12]. The trial was conducted between March 2019 and July 2020.

A mixed-method process evaluation was nested within the IMPULSE trial, using the United Kingdom Medical Research Council process evaluation of complex intervention guidance [13]. The objectives of the process evaluation were to explore the context influencing the implementation of the DIALOG+ intervention, intervention fidelity, and acceptability of the intervention to patients and clinicians [14].

### Settings and participants

In total, 11 outpatient mental health services across five countries (three in Montenegro and two in each of the other four countries) participated in the trial. Clinicians were eligible if they had a professional qualification and more than 6 months’ experience working in mental health care. The clinicians’ caseloads were screened to identify eligible patients aged 18 years or older, primary diagnosis of psychosis in remission (ICD-10 F20–29, F31) [15], attending the outpatient clinic or day hospital, lifetime history of at least one hospital admission, and capacity to provide informed consent. Exclusion criteria included diagnosis of organic brain disorders and severe cognitive deficits thus being unable to provide information to study instruments. Written informed consent was obtained from all clinicians and patients. The study received approvals from institutional research ethics committees.

### Intervention arm

The intervention arm received DIALOG+ during routine clinical appointments instead of usual care. The DIALOG+ intervention was offered to patients six times over 12 months (once per month for the first 3 months and then once every 3 months). The main features of the DIALOG+ intervention have been outlined in the Introduction. Each DIALOG+ session begins with patients’ self-assessment on a tablet computer [10]. The patients assess their satisfaction with eight life areas (mental health, physical health, job situation, accommodation, leisure activities, friendships, relationship with family/partner and personal safety) and three treatment areas (medication, practical help and meetings with professionals). Satisfaction with each area is rated on a 1—(totally dissatisfied) to 7—(totally satisfied) point scale. Next, the patient and clinician both reflect on the patient’s concerns, potentially comparing the current ratings with any previous ones to see which domains the patient has improved or deteriorated. Patients are then invited to choose an area they wish to discuss further during the meeting. A four-step, solution-focused approach (Understanding, Looking Forward, Exploring Options, and Agreeing on Actions) is used as a discussion guide to recognize and apply the patient’s existing resources. The session concludes with the patient and the clinician agreeing on actions that the patient, clinician and/or another person from the patient’s life should accomplish before the next meeting to improve the patient’s life and treatment satisfaction [10].

The first session should last up to 60 min and follow-up sessions up to 30 min [10]. The offered intervention always has the same format and clinicians use the same technique in each session. The content of sessions varies as per patients’ choice, for example, patients could choose to discuss different life domains in each session.

### Control arm

The control arm received standard care which includes routine clinical appointments, following the same delivery schedule as the intervention arm. These appointments generally focused on reviewing prescribed medication (e.g., is the medication effective, should the dose be modified, are there any side effects), discussing other aspects of care (e.g., referral to psychological therapy or social care), and providing general support (e.g., reassuring the patient stressed due to experiencing derogatory auditory hallucinations) [12]. At the beginning of the study, clinicians were asked not to change their usual clinical approach. The trial process evaluation included audiotaping and analyzing sessions from both arms [14]. The purpose of this work was first to assess contamination and differentiation between sessions in both arms. Secondly, we wanted to improve our understanding of the content and form of control sessions.

### Implementation strategy

The implementation strategy is a data-driven and theory informed document that was created before the trial to guide implementation of DIALOG+ [16]. The strategy was based on data from interviews and focus groups with stakeholders conducted prior to implementation of the intervention. The strategy provides detailed descriptive information of each potential barrier and the actions and resources to overcome it. For example, during pretrial work the most common barrier was clinicians’ perceived lack of capability and skills to deliver DIALOG+ to patients with psychosis. As a potential solution, DIALOG+ training was modified: training materials were adapted and appropriate behavior change techniques were used to classify active ingredients of the intervention and to address the identified barriers to implementation [16, 17]. Each clinician in the intervention arm received face-to-face training by a local research team member before the first DIALOG+ session, followed by top-up training after delivering the third session. Each clinician was able to access individual supervision provided by the study researchers after each session.

### Outcome measures

#### Aim 1

The primary outcome measure was quality of life (Manchester Short Assessment of Quality of Life (MANSA) [18]) at 12-months postrandomization. The secondary outcome measures were mental health (Brief Psychiatric Rating Scale [BPRS] [19], Clinical Assessment Interview for Negative Symptoms [CAINS] [20], and Brief Symptom Inventory [BSI] [21]); satisfaction with services (Client Satisfaction Questionnaire [CSQ-8] [22]); and economic costs at 12-months post-randomization. The cost-effectiveness analysis will be published separately. Two additional measures of quality of life (Re-QoL-10 [23] and EQ-5D-5L [24]) were used for the economic evaluation. With exception of the CAINS that was administered at baseline and 12 months, all other measures were administrated at baseline, at 6 months (after four DIALOG+ sessions) and at 12 months (after six DIALOG+ sessions).

#### Aim 2

The intervention fidelity was operationalized as adherence to the intervention protocol in terms of frequency, duration, content, and coverage [11]. Measures included survey after each session conducted by unblinded researchers, sourcing data from the clinician and/or the tablet, and audiotapes of sessions upon clinician and patient consent [12]. For this trial, high intervention fidelity was defined as follows: more than 80% of patients receiving a maximum (six) sessions of the DIALOG+ intervention (*frequency*), DIALOG+ sessions lasting at least 20 min (*duration*), good adherence to the DIALOG+ manual defined as total DAS score above 12 (*content*), and good retention in the trial defined as 80% for clinicians and patients, respectively (*coverage*).

### Impact of the COVID-19 pandemic

The COVID-19 pandemic was announced in March 2020 when the trial was in its final phase. The delivery of fifth and sixth DIALOG+ sessions and 12-month patient assessments had to be conducted remotely. This led to substantial changes in the intervention, patient assessments, and data collection. The patient–clinician pairs were not able to meet in person or use the tablet together. Researchers were not able to complete researcher-observed ratings on BPRS [19] and CAINS [20], so only patient-reported items were collected and analyzed for these scales. Furthermore, patients’ responses to the questionnaires could have been influenced by distress and lack of social support during pandemic. All of this made 12-month data difficult to interpret. The IMPULSE consortium agreed to focus on outcomes at 6-months post-randomization which was approved by the Scientific Advisory Board.

### Randomization and blinding

Eligible clinicians and patients were identified and consented. Once a patient gave informed consent, researchers completed the baseline assessment which included sociodemographic, clinical characteristics, and outcomes measures as listed above. To minimize selection bias within clusters, clinicians were randomized once all patients from their caseload had been recruited and all baseline assessments completed. Clinicians were randomized 1:1 to intervention or control. Randomization was conducted by an independent statistician who used sequential computer-generated random numbers to determine allocation. To prevent an unequal allocation across groups, participants were stratified before randomization on two factors: gender (e.g., male vs. female) and professional status (e.g., psychiatrists vs. nonpsychiatrists). Owing to the nature of the intervention, clinicians and patients could not be blinded to their allocation. Researchers who conducted the assessments were blinded to the allocation of patients.

### DIALOG+ adherence scale

The DIALOG+ adherence scale (DAS) was developed by DIALOG+ experts at Queen Mary University of London. The scale is used to assess clinician behaviors specific to the delivery of DIALOG+ (e.g., selection of areas for further discussion, the four-step approach) [25] as specified in the manual [10]. The scale comprises 19 items, scored using a two-point scale (0, 1) to indicate either the absence or implementation of a specific behavior. There are three subscales:Adherence to the initial DIALOG scale and review of ratings (seven items), Adherence to the four-step approach (nine items), and Quality of interaction (three items). A mean score (min, max) for each item, three subscales, and the total score was calculated across the sample. Structured analysis of audiotapes from 17 clinicians from the intervention arm was used to assess clinicians’ Adherence to the DIALOG+ manual. Audiotaped control sessions were also assessed against the DAS to evaluate intervention differentiation and contamination. A higher score indicates a better clinician’s Adherence to the DIALOG+ manual [10]. Similar DAS scores in both arms would indicate that clinical work was not substantially different and that clinical appointments in the control arm could have been contaminated.

### Sample size

Preliminary sample size calculations were based on the data from the original DIALOG+ trial in which the effect size was 0.35 [8]. This effect size reflected improved quality of life ratings for five of 12 life domains in the DIALOG+ intervention, which is considered clinically and socially relevant. To detect an effect size of 0.35, this trial needed 260 patients. The number was inflated to account for cluster design and 10% dropout of clinicians. The intracluster correlation coefficient (ICC) was 0.05, and the design effect due to cluster randomization was 1.2 [26]. Assuming a standard deviation of 1.0, with 80% power at 5% significance level, the study required a minimum of 36 clinicians in each group (72 in total). To allow for equal number of clinicians per country, the study aimed to recruit a minimum of 16 clinicians per country (eight in the intervention arm, eight in the control arm). In total, the study aimed to recruit 80 clinicians and 400 patients.

### Data analysis

The randomization of patients was performed at the cluster level, so there were potential imbalances in patient characteristics between arms. Therefore, the first analyses compared characteristics of the arms at baseline. The outcome measures were compared between arms at both 6- and 12-months based on observed data only. To allow for the clustering of data into different sites and different clinicians, analysis was performed using multilevel regression methods. Three-level models were utilized. These considered measurement from individual patients contained within measurements from the same clinician. These in turn were nested within the individual sites (i.e., countries). The mean difference in outcome between arms, indicative of the magnitude of the effect, was calculated as outcome for the intervention arm minus outcome in the control arm and presented along with corresponding confidence intervals. The arm difference was adjusted for outcomes at baseline as well as for other covariates included in the models, including patient age, patient diagnosis, and clinician profession (psychiatrist vs. non-psychiatrist).

Analyses were performed on an intention-to-treat basis, including all randomized patients. To allow for missing data, multiple imputation methods were used in a sensitivity analysis (Supplementary Appendix I). Subgroup analyses examined data from Serbia only, as this was the only country to complete the trial before the COVID-19 restrictions. Two-level multilevel models were used as all data came from the same country (Supplementary Appendix II). A p-value less or equal to 0.05 was considered statistically significant.

## Results

### Participants

**Figure 1.**
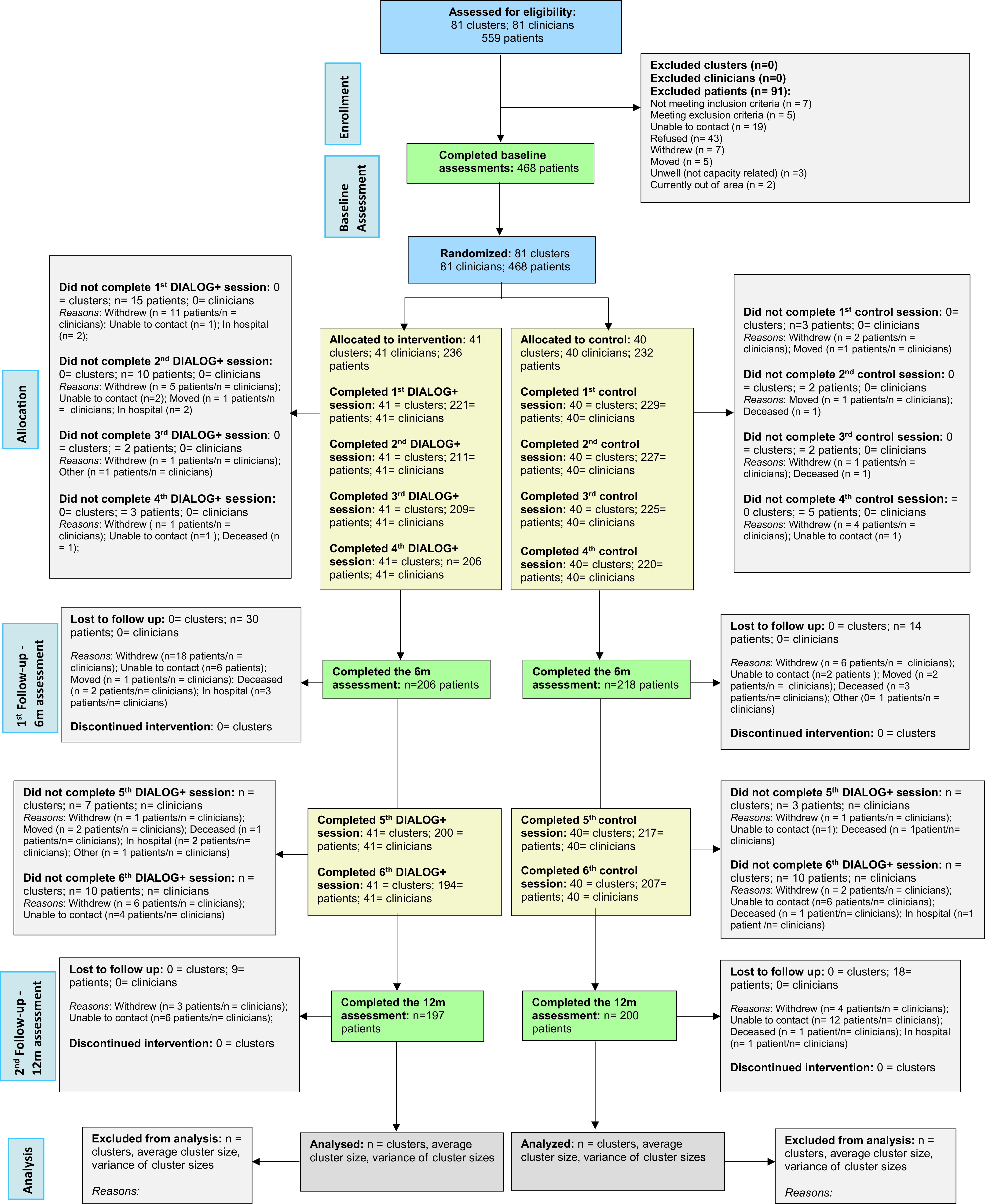
IMPULSE trial CONSORT for cRCTs.

A total of 81 clinicians and 468 of their patients were randomized to either DIALOG+ (intervention) or standard care (control). Figure 1 shows a CONSORT diagram summarizing the participant flow. All clinicians were retained for the duration of the trial, while 31 patients withdrew from the study (21 in the intervention arm), 34 were unable to be contacted/moved/hospitalized at follow-up points (16 in the intervention arm), and six died during the trial (two in the intervention arm from causes unrelated to the intervention/research methods).

Table 1 summarizes the characteristics of the patients and clinicians. Compared to the control arm, the intervention arm patients were older, had a higher proportion of schizophrenia diagnoses, and had fewer patients with previous psychological treatment.

**Table 1.** Patients’ and clinicians’ characteristics at baseline.

		Standard care (control)		DIALOG+(intervention)		
		*N*	Summary^a^	*N*	Summary^a^	Statistics
Patients						
Country (all patients)	Total	232		236		
	Bosnia and Herzegovina		41 (17.7%)		40 (17.0%)	N/A^b^
	Macedonia		41 (17.7%)		41 (17.4%)	
	Kosovo*		51 (22.0%)		52 (21.0%)	
	Montenegro		60 (25.9%)		62 (26.3%)	
	Serbia		39 (16.8%)		41 (17.4%)	
Age	—	232	40.8 ± 11.3	236	44.3 ± 11.1	**0.005**
Sex	Female	232	111 (47.8%)	236	103 (43.6%)	0.55
	Male		121 (52.2%)		133 (56.4%)	
Marital status	Single	232	133 (57.3%)	236	121 (51.3%)	0.22
	Married/cohabitating		59 (25.4%)		66 (28.0%)	
	Separated/divorced		37 (16.0%)		38 (16.1%)	
	Widow/widower		3 (1.3%)		11 (4.7%)	
Level of education	Less elementary	232	7 (3.0%)	236	2 (0.9%)	0.33
	Elementary		30 (12.9%)		49 (20.8%)	
	High school		144 (62.1%)		139 (58.9%)	
	University		45 (19.4%)		40 (17.0%)	
	Postgraduate		4 (1.7%)		4 (1.7%)	
	Other		2 (0.9%)		2 (0.9%)	
Living situation	Living alone	232	29 (12.5%)	236	30 (12.7%)	0.472
	With others		207 (89.2%)		202 (85.6%)	
ICD-10 diagnosis	Schizophrenia	232	129 (55.6%)	236	155 (65.7%)	**0.03**
	Bipolar disorder		43 (18.5%)		24 (10.2%)	
	Other diagnosis		60 (25.9%)		57 (24.2%)	
Antipsychotic medication^c^	—	202	9.9 (6.6)	213	11.9 (6.8)	**0.02**
Number of hospitalizations	—	228	1 [1, 4]	231	2 [1, 4]	0.33
History of receiving psychological treatment	No	231	112 (48.5%)	232	141 (60.8%)	**0.03**
	Yes		119 (51.5%)		91 (39.2%)	
Clinician’s sex	Female	232	175 (75.4%)	236	189 (80.1%)	0.21
	Male		57 (24.6%)		47 (19.9%)	
Clinician’s profession	Psychiatrists	232	174 (75.0%)	236	145 (61.4%)	**<0.001**
	Other profession		58 (25.0%)		91 (38.6%)	
Clinicians						
Country (all clinicians)	Total	40		41		N/A^b^
	Bosnia and Herzegovina		8 (20%)		8 (19.5%)	
	Macedonia		8 (20%)		8 (19.5%)	
	Kosovo*		8 (20%)		8 (19.5%)	
	Montenegro		8 (20%)		8 (19.5%)	
	Serbia		8 (20%)		9 (22%)	
Clinician’s sex^d^	Female	61	28 (45.9%)		33 (54.1%)	0.202
	Male	20	12 (60%)		8 (40%)	
Clinician’s profession^d^	Psychiatrists	55	28 (50.9%)		27 (49.1%)	0.44
	Other profession	26	12 (46.7%)		14 (53.8%)	

a *Summary statistics are number (percentage) or mean ± standard deviation or median [interquartile range].*

b N/A—No formal comparison between arms made, as country included as random effect in multilevel model.

c Daily antipsychotic dosage was calculated and converted into olanzapine equivalents [27, 28]. Chlorpromazine equivalent dose of 400 mg/day corresponds to 13.2 mg OLA equivalents.

d Reported as gender/profession of each patient’s clinician.

### Effectiveness

Table 2 shows primary and secondary outcome comparisons at 6- and 12-months post-randomization. There was a statistically significant difference between intervention and control arms. At 6 months, MANSA scores were significantly higher (reflecting better quality of life) in the intervention arm (4.84 ± 0.98 vs. 4.65 ± 0.97, 95% CI 0.18 [0.01, 0.35], *p* = 0.03). The improvement was the largest in three MANSA subscales, namely Friendships (4.87 ± 1.60 vs. 4.40 ± 1.75, 95% CI 0.45 [0.13, 0.78], *p* = 0.006), Accommodation (5.63 ± 1.42 vs. 5.39 ± 1.44, 95% CI 0.27 [0.02, 0.51], *p* = 0.03, and Living situation (5.67 ± 1.34 vs. 5.49 ± 1.55, 95% CI 0.29 [0.03, 0.54], *p* = 0.03). The differences in scores are indicative of small effect size on MANSA total, MANSA Accommodation and MANSA Living situation. Regarding MANSA Friendships, the difference in scores equates to a standardized effect size of approximately 0.45 units which is indicative of medium effect size.

**Table 2.** Primary and secondary outcomes at baseline, 6 and 12 months.

	Standard care (control)		DIALOG+ (intervention)		Difference	
Outcome	*N*	Mean ± SD	*N*	Mean ± SD	Mean (95% CI)	Statistics^a^
Quality of Life (MANSA)						
Baseline	232	4.54 ± 0.96	236	4.48 ± 0.95	0	
6 months	218	4.65 ± 0.97	206	4.84 ± 0.98	0.18 (0.01, 0.35)	**0.03**
12 months	201	4.86 ± 1.04	196	4.94 ± 1.02	0.14 (−0.04, 0.32)	0.12
Observed clinical symptoms (BPRS)						
Baseline	231	1.77 ± 0.48	234	1.79 ± 0.54	0	
6 months	214	1.58 ± 0.46	203	1.54 ± 0.46	−0.05 (−0.14, 0.03)	0.21
12 months	117	1.53 ± 0.50	99	1.59 ± 0.55	0.01 (−0.12, 0.14)	0.90
Self-reported mental health problems (BSI)						
Baseline	232	0.98 ± 0.70	236	1.03 ± 0.78	0	
6 months	218	0.83 ± 0.71	206	0.82 ± 0.67	−0.02 (−0.11, 0.07)	0.59
12 months	200	0.77 ± 0.70	196	0.81 ± 0.70	−0.01 (−0.10, 0.09)	0.88
Treatment satisfaction (CSQ-8)						
Baseline	232	26.9 ± 4.7	236	27.3 ± 4.5	0	
6 months	218	27.6 ± 3.9	206	28.6 ± 3.6	0.6 (−0.2, 1.3)	0.13
12 months	201	28.5 ± 4.0	196	28.9 ± 3.6	0.2 (−0.5, 1.0)	0.59
Negative symptoms (CAINS-MAP)^a^						
Baseline	225	15.8 ± 8.3	228	16.2 ± 9.0	0	
6 months	—	—	—	—	—	—
12 months	193	13.9 ± 9.0	182	14.1 ± 9.0	−0.1 (−1.6, 1.4)	0.86
Negative symptoms (CAINS-EXP)^b^						
Baseline	232	1.9 ± 2.0	236	2.2 ± 2.0	0	
6 months	—	—	—	—	—	—
12 months	198	1.32 ± 1.78	185	1.59 ± 1.82	−0.03 (−0.34, 0.29)	0.87
Quality of life (EQ-5D-5L)						
Baseline	232	0.93 ± 0.13	235	0.89 ± 0.16	0	
6 months	218	0.93 ± 0.12	206	0.93 ± 0.13	0.01 (−0.01, 0.04)	0.30
12 months	201	0.95 ± 0.09	196	0.93 ± 0.12	0.00 (−0.02, 0.02)	0.81
Recovering quality of life (ReQoL-10)						
Baseline	232	25.7 ± 8.5	236	25.7 ± 8.1	0	
6 months	218	26.2 ± 8.3	206	27.2 ± 7.9	0.7 (−0.5, 1.9)	0.23
12 months	201	27.8 ± 8.7	196	27.7 ± 8.3	0.2 (−1.0, 1.4)	0.71

a *Differences calculated as outcomes for DIALOG+minus values for standard care. Differences adjusted for outcome values at baseline, patient age, diagnosis, and clinician type.*

b CAINS was administered only at baseline and 12 months.

The secondary outcomes at 6 months were not significantly different between arms.

### Sensitivity analysis

Only Serbia completed data collection before pandemic restrictions were introduced locally. The analysis of Serbian data at the 6-month time point indicated no significant difference between arms for the MANSA, BPRS, and BSI total scores. The intervention arm scored significantly higher on the CSQ-8 scale, indicating higher treatment satisfaction. The results at 12 months show significantly higher quality of life in the intervention arm (MANSA total score, 5.18 ± 0.86 vs. 4.42 ± 0.65, 95% CI 0.55 [0.18, 0.92], *p* = 0.004). The CSQ-8 score was also significantly higher at 12 months in the intervention arm (29.2 ± 2.9 vs. 26.3 ± 4.2, 95% CI 2.0 [0.6, 3.5], *p* = 0.007). No significant difference was observed for other measures. Results are shown in Supplementary Appendix II. To allow for missing data, multiple imputation methods was used and, as shown in Supplementary Appendix I, results do not differ significantly compared with results from observed data only. This subsample was not powered to detect the expected effect size and, therefore, reported findings are of explorative nature and indicative of what could have been found if the trial was not disrupted by the pandemic.

### Intervention fidelity and differentiation between the intervention and control arms

Table 3 shows characteristics of clinical appointments in both arms with emphasis on intervention fidelity and differentiation between the intervention and control.

**Table 3.** Intervention fidelity and differentiation.

Intervention fidelity				
Measures of adherence	Indicators from the IMPULSE trial		Standard care (control) *N* = 232	DIALOG+(intervention) *N* = 236
*Frequency:* The study protocol includes six sessions per patient	Planned sessions, *N* (%)		1,392 (100)	1,416 (100)
	Delivered sessions, *N* (%)		1,325 (95.2)	1,241 (87.6)
	Number of sessions per patient, mean (SD)		5.7 (2.4)	5.3 (1.8)
	Patients receiving six sessions, *N* (%)		207 (89.2)	194 (82.2)
	Patients receiving four sessions, *N* (%)^a^		220 (94.8)	206 (87.3)
*Duration:* The intervention manual suggests up to 60 min for the first session and up to 30 min for the follow-up sessions	Duration of sessions in minutes, mean (SD)		20.1 (11.2)	28 (11.8)
	Duration of first session in minutes, mean (SD)		20.5 (10.2)	23.8 (12.7)
	Duration of follow-up sessions in minutes, mean (SD)		20.2 (12.1)	28.2 (11.3)
	Sessions lasting ≥20 min, *N* (%)^b^		611 (46.1)	969 (78.1)
*Content:* The intervention manual suggests: (a) rating patient satisfaction with 11 domains (DIALOG scale), (b) identifying three domains for further discussion, (c) going to the four-step approach to address concerns, and (d) set at least one action. Assessment using the DIALOG+ adherence scale (DAS)	DAS score	Adherence to the initial DIALOG scale and review of ratings, mean (SD) (range)	0.8 (1)	4.4 (1.2)
			(0–7)	(0–7)
		Adherence to the four-step procedure, mean (SD) (range)	1.5 (2.3)	6.9 (1.8)
			(0–9)	(0–9)
		Quality of interaction, mean (SD) (range)	1.3 (0.7)	2.7 (0.4)
			(0–3)	(0–3)
		Total score, mean (SD) (range)^c^	3.5 (3.6)	13.9 (2.6)
			(0–19)	(0–19)
	Selected domains per session, mean (SD)		N/A	1.8 (0.9)
	Sessions with three domains selected, *N* (%)		N/A	310 (25)
	Number of set actions, mean (SD)		N/A	2.52 (1.4)
	Number of meetings with at least one action set, *N* (%)		N/A	1,232 (99.3)
*Coverage:* Defined as whether all the people who should be receiving the intervention actually do so. The protocol suggests that all clinicians and all patients remain involved in the intervention over 12 months	Clinicians’ retention in the trial^d^		40 (100)	41 (100)
	Patients’ retention in the trial^d^		197 (84%)	200 (85%)
*The IMPULSE trial defined high intervention fidelity as:* ^a^Min.80% of patients receiving at six sessions of DIALOG+ (frequency) ^b^Sessions lasting at least 20 min (duration) ^c^Good adherence to the DIALOG+ manual defined as total mean DAS score above 12 (*content*) ^d^Good retention in the trial defined as 80% for clinicians and patients, respectively (*coverage*)			The majority of patients received sessions with frequency and duration as planned per protocol. Adherence to content was assessed in reference to DIALOG+ sessions indicating intervention differentiation.	The majority of patients received DIALOG+ sessions with frequency and duration as planned per protocol. Adherence to content was good with variability in initial review of domains and selecting at least three for further discussion. All clinicians and 85% patients were retained in the trial over 12 months

*Note:* DAS stands for DIALOG+Adherence scale [25], ratings have been obtained from 37 audio-recordings of sessions (17 from the DIALOG+sessions).

As per the protocol, the trial aimed to deliver six sessions per patient or 2,808 sessions in total. The mean number of sessions was 5.7 (SD = 2.4) in the control and 5.3 (SD = 1.8) in the intervention arm. In total 194 participants (82.2%) received all six planned DIALOG+ sessions. We found that 206 participants (87.3%) received four DIALOG+ sessions which is relevant because the intervention was found effective at 6 months/after four sessions. The mean duration of sessions was 20.1 min (SD = 11.2) in the control and 28 min (SD = 11.8) in the intervention arm.

Based on DAS, adherence to the initial DIALOG scale and review of ratings was good (mean = 4.4, range 0–7, SD = 1.2). Adherence to the four-step approach was very good (mean = 6.9, range 0–9, SD = 1.8). The mean number of selected domains per session was 1.8 (SD = 0.9). In 25% (*N* = 310) of sessions, patient–clinician pairs selected three domains as suggested in the manual. In 48.8% (*N* = 606) of sessions, one domain was selected, and in 73.2% (*N* = 908) of sessions two domains were selected. In 99.3% of sessions (*N* = 1,232) at least one action was agreed between the patient and the clinician. Overall, the clinicians’ adherence to the DIALOG+ manual [10] was good (mean 13.9, SD 2.6, range 0–19). The DAS score of recording in the control arm (mean 3.5, SD 3.6, range 0–19) indicate good differentiation between the two arms in regards to content of delivered clinical appointments. This finding also indicates that clinical appointments in the control arm were not contaminated.

In conclusion, the intervention was delivered with high fidelity. In total 85% of patients received maximum number of DIALOG+ intervention (*frequency*). On average DIALOG+ sessions lasted 20 min (*duration*). Adherence to content was good with variability in initial review of domains and selecting at least three for further discussion (DAS total mean 13.9, SD 2.6) (*content*). The trial had good retention of clinicians (100%) and patients (85%), respectively (*coverage*).

## Discussion

### Main findings

This study showed that a digital psychosocial intervention such as DIALOG+ can be implemented in mental health services in SEE with high fidelity. Clinical appointments in both arms did not differ in the type of clinical setting or frequency, although appointments in the intervention arm lasted on average 8 min longer. The trial results show that DIALOG+ improved subjective quality of life of individuals with psychosis at 6 months (four sessions), albeit with small effect size. Medium size effect was found regarding improved satisfaction with friendships in the intervention arm. There was no effect on clinical symptoms. Because the trial was disrupted by the COVID-19 pandemic, the effect of the complete intervention at 12 months (after six sessions) could not be explored. Findings from a country which completed data collection before the COVID-19 pandemic were indicative of improved subjective quality of life after 12 months (six sessions).

### Strengths and limitations

This is the largest ever nonpharmacological RCT involving individuals with psychosis conducted in SEE, and among few such trials globally. The intervention was delivered with exceptionally high fidelity, thus ensuring that the observed effect can be attributed to the DIALOG+ intervention. The effect size was small; however, we believe this is still clinically relevant because the field of mental health care lacks interventions focused on improving quality of life of individuals with psychosis [29]. The intervention was effective after four sessions which, compared to more intensive and resource-demanding interventions, increases the relevance of these findings for clinical practice. Due to the pragmatic nature of the trial, results can be generalized and applied in routine practice settings. A further strength was the hybrid design and combination of clinical effectiveness and implementation science to enhance impact [30]. The trial was guided by implementation strategy developed using the data from the pilot stage and informed by behavioral theory, which is often neglected in the intervention literature.

The study also has several limitations. Clinicians could not be blinded toward their own allocation, which raises the possibility of performance bias. The increased time spent on average between patient and clinicians in the intervention arm could itself have had an effect on improved patients’ outcomes. The cluster randomization minimized the risk of contamination compared to individual randomization. The randomization at the service/provider level would have minimized the risk further; however, this was not possible due to practical constraints. Because the study was affected by the COVID-19 pandemic, only outcomes at 6-months post-randomization were analyzed across five countries. It is worth noting that our data from Serbia as well as trial data with patients with depression and anxiety conducted in Bosnia and Herzegovina [31] indicate that treatment including DIALOG+ is beneficial at 12 months post-randomization.

### Interpretation of trial findings

DIALOG+ now has strong evidence base for improving quality of life of patients with psychosis, which is an important patient-reported outcome measure. The findings of this trial partially replicate the original DIALOG+ trial in the UK [8], in that there was a positive effect of the intervention on patients’ quality of life in both trials. However, in this SEE trial, the intervention had no effect on clinical outcomes or satisfaction with services, while in the UK trial patients in the intervention arm has significantly fewer unmet needs, fewer symptoms, and better objective social outcomes [8]. Patients in both trials were similar in regards to most sociodemographic and clinical characteristics. However, there were some differences, the IMPULSE trial had more female participants (46% vs. 31% in the UK trial) and more participants diagnosed with affective psychosis (15% vs. 5% in the UK trial). Regarding the main outcome measure (MANSA), patients in the IMPULSE trail had higher baseline scores compared to patients in the UK trial, indicating that there was less room for improvement with the intervention in the IMPULSE trial. Patients in the IMPULSE trial were in remission, which was defined based on treating clinician’s judgment. This was not eligibility criteria for the UK trial, however, since they recruited outpatients treated in the community more than 1 month, it could be expected that the majority would not be acutely unwell to warrant hospital admission.

In this SEE trial, the intervention was exceptionally well implemented; only 6% of participants did not receive the intervention and the average number of delivered DIALOG+ sessions was 5.3 (SD = 1.8; maximum was six sessions). In the UK trial [8], the implementation was significantly more variable; 30% of patients did not receive the intervention. Patient groups in both trials were similar in regards to their sociodemographic and clinical characteristics. There are several possible explanations for the good implementation and engagement from both clinicians and patients in the SEE trial. First, joint pretrial work on the implementation strategy allowed us to address key barriers to implementation in advance. Second, 60% of patients in the intervention arm had never received any psychological treatment, so they potentially perceived DIALOG+ as a positive change or novelty in their mental health care. Similarly, the majority of clinicians in the participating countries were not trained to deliver psychosocial interventions, so they welcomed training and improvement of their skillset. Finally, due to almost complete lack of mental health research studies in SEE, patients and clinicians appreciated being included in an international research project.

### Future implications

Although most international guidelines suggest a combined-therapy approach including antipsychotic medication, talking therapy, and family support, most research in this area is still focused on advances in pharmacotherapy. Previous reports indicate that effective psychosocial interventions are rarely offered to individuals with psychosis [7, 32]. Possible explanations include the lack of effective interventions, difficulties in clinical engagement and retention in services, and failure to implement and sustain effective interventions in routine practice [33].

This study shows that many of these barriers could be overcome with an intervention which can be incorporated into already existing routine clinical appointments. Despite low national mental health care budgets in participating countries [6], patients with psychosis are regularly seen within secondary mental health care services. They are offered appointments which are an excellent opportunity to introduce DIALOG+, thus making the appointments themselves therapeutically more effective. The preliminary findings of the cost-effectiveness analysis indicate the cost-saving potential of the intervention in several participating countries (full analysis will be published separately). The next step following this trial would be to consider wider implementation of DIALOG+ while ensuring its sustainability across mental health services.

This study has provided useful insights into conducting effectiveness–implementation research in LMICs. Throughout the duration of the study, approximately 50 researchers across five LMICs were trained in mental health research methodology and stakeholder engagement. More than 100 clinicians were trained to deliver psychosocial treatment to individuals with psychosis. It is reasonable to expect that these researchers and clinicians will contribute to future research and innovation in the field.

## Conclusion

The DIALOG+ intervention has an emergent evidence base for how to improve the quality of life of patients with psychosis. The study adds to evidence on effective treatments for individuals with psychosis and on implementation of interventions in routine practice settings. This is particularly relevant for SEE countries due to lack of research studies and understanding of implementation barriers and facilitators. The effective and generic intervention have the potential to contribute to closing the treatment gap for psychosis and expanding access to care for this clinical population.

## Data Availability

The data that support the findings of this study are available from the corresponding author upon reasonable request.

## Supplementary material

For supplementary material accompanying this paper visit http://doi.org/10.1192/j.eurpsy.2022.2302. click here to view supplementary material
