# Supplementary material for: Improving treatment of patients with psychosis in low-and-middle-income countries in Southeast Europe: Results from a hybrid effectiveness-implementation, pragmatic, cluster-randomized clinical trial (IMPULSE)
Source: Eur Psychiatry. 2022 Aug 10;65(1):e50. doi: 10.1192/j.eurpsy.2022.2302 (PMC9491080; doi:10.1192/j.eurpsy.2022.2302)
Supplement: Supplementary file 1 [file epasup.zip › S0924933822023021sup001.docx]

**Appendix I –** Outcomes at 6 and 12 months - Multiple Imputation approach

| Outcome | 6 months | | 12 months | |
| --- | --- | --- | --- | --- |
|  | Mean Difference (95% CI)^*^ | Statistics | Mean Difference (95% CI) ^a^ | Statistics |
|  |  |  |  |  |
| MANSA total | 0.17 (0.00, 0.33) | 0.06 | 0.10 (-0.07, 0.27) | 0.26 |
|  |  |  |  |  |
| MANSA whole life | 0.12 (-0.16, 0.39) | 0.42 | 0.11 (-0.17, 0.39) | 0.45 |
| MANSA job | 0.12 (-0.22, 0.46) | 0.49 | 0.08 (-0.30, 0.47) | 0.68 |
| MASNA financial | 0.18 (-0.12, 0.48) | 0.24 | -0.08 (-0.45, 0.28) | 0.66 |
| MANSA friendship | 0.42 (0.09, 0.75) | **0.01** | 0.26 (-0.07, 0.60) | 0.12 |
| MANSA accommodation | 0.21 (-0.04, 0.46) | 0.10 | 0.10 (-0.18, 0.37) | 0.49 |
| MANSA safety | 0.19 (-0.08, 0.46) | 0.18 | 0.11 (-0.16, 0.38) | 0.44 |
| MANSA living | 0.25 (-0.02, 0.52) | 0.07 | 0.21 (-0.06, 0.49) | 0.12 |
| MANSA sex | -0.02 (-0.38, 0.34) | 0.92 | 0.11 (-0.27, 0.49) | 0.56 |
| MANSA family | 0.06 (-0.23, 0.34) | 0.69 | 0.08 (-0.22, 0.39) | 0.59 |
| MANSA physical health | 0.02 (-0.27, 0.31) | 0.90 | 0.03 (-0.27, 0.33) | 0.84 |
| MANSA mental health | 0.21 (-0.06, 0.48) | 0.12 | 0.13 (-0.19, 0.44) | 0.42 |
|  |  |  |  |  |
| BPRS total | -0.04 (-0.12, 0.04) | 0.37 | 0.01 (-0.10, 0.11) | 0.91 |
| BPRS anxiety/depression | -0.09 (-0.26, 0.07) | 0.28 | -0.05 (-0.23, 0.14) | 0.62 |
| BPRS positive | 0.00 (-0.12, 0.12) | 0.97 | 0.08 (-0.06, 0.22) | 0.28 |
| BPRS negative | -0.14 (-0.33, 0.04) | 0.13 | -0.05 (-0.26, 0.15) | 0.62 |
| BPRS hostility | -0.02 (-0.12, 0.07) | 0.65 | -0.03 (-0.15, 0.09) | 0.17 |
| BPRS activation | -0.05 (-0.16, 0.06) | 0.40 | 0.01 (-0.17, 0.20) | 0.90 |
|  |  |  |  |  |
| BSI total | -0.02 (-0.12, 0.07) | 0.65 | 0.01 (-0.09, 0.12) | 0.80 |
| CAINS – MAP^b^ | - |  | 0.0 (-1.5, 1.5) | 0.99 |
| CAINS – EXP^b^ | - |  | -0.02 (-0.38, 0.34) | 0.91 |
| CSQ-8 | 0.6 (-0.2, 1.3) | 0.14 | 0.3 (-0.5, 1.1) | 0.50 |
| EQ-5D-5L | 0.01 (-0.01, 0.04) | 0.29 | 0.00 (-0.02, 0.02) | 0.79 |
| ReQoL-10 | 0.7 (-0.5, 1.9) | 0.25 | 0.1 (-1.2, 1.4) | 0.86 |

*Note.* ^a^Differences calculated as outcomes for DIALOG+ minus values for Standard care. Differences adjusted for outcome values at baseline, patient age, diagnosis and clinician type. ^b^CAINS was administered only at baseline and 12 months.
