# Supplementary material for: Improving treatment of patients with psychosis in low-and-middle-income countries in Southeast Europe: Results from a hybrid effectiveness-implementation, pragmatic, cluster-randomized clinical trial (IMPULSE)
Source: Eur Psychiatry. 2022 Aug 10;65(1):e50. doi: 10.1192/j.eurpsy.2022.2302 (PMC9491080; doi:10.1192/j.eurpsy.2022.2302)
Supplement: Supplementary file 1 [file epasup.zip › S0924933822023021sup002.docx]

**Appendix II –** Key outcomes at 6 and 12 months for participants from Serbia

| Outcome | Standard care  (control arm) | | DIALOG+  (intervention arm) | | Difference ^a^ | Statistics |
| --- | --- | --- | --- | --- | --- | --- |
|  | N | Mean ± SD | N | Mean ± SD | Mean (95% CI) |  |
| **Quality of Life (MANSA)** |  |  |  |  |  |  |
| Baseline | 36 | 4.16 ± 0.59 | 37 | 4.48 ± 0.74 | 0 |  |
| 6 months | 36 | 4.42 ± 0.71 | 37 | 4.93 ± 1.09 | 0.23 (-0.12, 0.59) | 0.19 |
| 12 months | 36 | 4.42 ± 0.65 | 35 | 5.18 ± 0.86 | 0.55 (0.18, 0.92) | **0.004** |
| **Observed clinical symptoms (BPRS)** |  |  |  |  |  |  |
| Baseline | 36 | 1.91 ± 0.44 | 37 | 1.81 ± 0.48 | 0 |  |
| 6 months | 36 | 1.77 ± 0.52 | 37 | 1.68 ± 0.45 | -0.02 (-0.22, 0.17) | 0.83 |
| 12 months | 21 | 1.65 ± 0.40 | 12 | 1.63 ± 0.38 | -0.07 (-0.30, 0.17) | 0.57 |
| **Self-reported mental health problems (BSI)** |  |  |  |  |  |  |
| Baseline | 36 | 1.18 ± 0.67 | 37 | 0.85 ± 0.64 | 0 |  |
| 6 months | 36 | 1.05 ± 0.66 | 37 | 0.83 ± 0.59 | 0.01 (-0.21, 0.23) | 0.92 |
| 12 months | 36 | 1.04 ± 0.69 | 35 | 0.75 ± 0.64 | -0.04 (-0.26, 0.19) | 0.74 |
| **Treatment satisfaction (CSQ-8)** |  |  |  |  |  |  |
| Baseline | 36 | 26.1 ± 3.3 | 37 | 27.3 ± 3.3 | 0 |  |
| 6 months | 36 | 26.3 ± 4.2 | 37 | 29.2 ± 2.9 | 2.0 (0.6, 3.5) | **0.007** |
| 12 months | 36 | 27.1 ± 4.3 | 35 | 28.8 ± 3.9 | 0.7 (-1.2, 2.7) | 0.44 |
| **Negative symptoms (CAINS-MAP)** ^b^ |  |  |  |  |  |  |
| Baseline | 36 | 19.6 ± 6.3 | 34 | 17.2 ± 8.5 | 0 |  |
| 6 months | - | - | - | - | - | - |
| 12 months | 36 | 17.3 ± 7.7 | 34 | 16.1 ± 7.9 | -0.5 (-4.7, 3.6) | 0.8 |
| **Negative symptoms (CAINS-EXP)** ^b^ |  |  |  |  |  |  |
| Baseline | 36 | 1.89 ± 1.53 | 34 | 2.38 ± 1.65 | 0 |  |
| 6 months | - | - | - | - | - | - |
| 12 months | 36 | 1.86 ± 1.90 | 34 | 1.97 ± 1.71 | -0.14 (-0.89, 0.62) | 0.72 |

*Note.* ^a^Differences calculated as outcomes for DIALOG+ minus values for Standard care. Differences adjusted for outcome values at baseline, patient age, diagnosis and clinician type. ^b^CAINS was administered only at baseline and 12 months.
